# Unraveling Intravascular Lobular Capillary Hemangioma: A Comprehensive Scoping Review

**DOI:** 10.7759/cureus.45142

**Published:** 2023-09-12

**Authors:** Abubakar Gapizov, Harmandeep K Sidhu, Maryam Affaf, Shamayel Safdar, Rabbia Irfan, Chukwuyem Ekhator, Muhammad Nabeel Saddique, Monika Devi, Elizabeth O Silloca Cabana, Muhammad Kamran, Sophia B Bellegarde, Iqbal Hussain

**Affiliations:** 1 Department of General Surgery, American University of Antigua, St. John's, ATG; 2 Department of Dermatology, Dayanand Medical College and Hospital, Ludhiana, IND; 3 Department of Medicine and Surgery, Indira Gandhi Medical College and Hospital, Nagpur, IND; 4 Department of Medicine and Surgery, Women Medical and Dental College, Abbotabad, PAK; 5 Department of Internal Medicine, Mayo Hospital, Lahore, PAK; 6 Department of Neuro-Oncology, New York Institute of Technology, New York, USA; 7 Department of Medicine and Surgery, Mayo Hospital, Lahore, PAK; 8 Department of Medicine, Ziauddin University, Karachi, PAK; 9 Department of Pathology, Wake Forest Baptist Health, Winston-Salem, USA; 10 Department of Pathology and Laboratory Medicine, American University of Antigua, St. John's, ATG; 11 Department of Internal Medicine, Khyber Medical University, Peshawar, PAK

**Keywords:** surgical excision, scoping review, vascular anomaly, lobular capillary hemangioma, intravascular lobular capillary hemangioma

## Abstract

This scoping review focuses on intravascular lobular capillary hemangioma (ILCH), a rare and distinct subset of lobular capillary hemangioma (LCH). This study provides a comprehensive overview of ILCH, delving into its clinical characteristics, origins, pathogenesis, diagnostic methods, treatment options, and outcomes. Despite its rarity, ILCH presents unique diagnostic and management challenges due to its intravascular origin. The review emphasizes the importance of accurate differentiation from other vascular lesions and underscores the need for histopathological confirmation. This article discusses the presentation of ILCH in the reported literature. The pathogenesis remains uncertain, with factors such as trauma, inflammation, hormonal changes, and medications being considered potential contributors. Histopathological features, imaging techniques, and diagnostic tools are discussed, highlighting the distinct histological architecture of ILCHs and the importance of immunohistochemical staining for accurate diagnosis. Surgical excision is the primary approach for managing ILCH due to its potential complications, including superior vena cava (SVC) occlusion and thrombosis. This review concludes by outlining potential directions for future research, including investigating genetic and molecular mechanisms underlying ILCH development, developing targeted therapies, building patient registries for collaborative efforts, and exploring minimally invasive surgical techniques. The importance of long-term patient outcome studies and international collaborations is emphasized to enhance our understanding of this rare vascular anomaly.

## Introduction and background

Intravascular lobular capillary hemangioma (ILCH), also referred to as pyogenic granuloma, is a benign vascular tumor that is typically localized within the blood vessels of the head, neck, and upper extremities [1,2]. The intravenous subtype of ILCH was initially documented by Cooper et al. in 1979 through a series of 18 cases [3]. The defining characteristic of pyogenic granuloma involves the excessive growth of vessel tissues into the vessel's interior and the invasion of surrounding tissues. Histopathological analysis reveals that this hemangioma type features clusters of capillaries enclosed by flattened endothelial cells within the upper skin layers, as well as dense clusters of robust-walled fibrovascular endothelial-lined vessels situated in the deeper skin layers [4].

The prevalence of ILCH has been observed across various age groups, encompassing both children and adults of both genders, with a slight prevalence in females. It is more commonly found during the second decade of life in young females, possibly due to hormonal influences [5]. However, discrepancies still exist regarding its exact occurrence. ILCH is widespread in locations such as the basilic vein, right subclavian vein, and iliac vein, taking the form of pyogenic granuloma, where the entire lesion presents as a solitary polyp-like mass projecting into a vein's lumen [6,7]. Furthermore, instances of this lesion have been reported in the oral mucosa and skin [8].

Although the precise cause of ILCH remains elusive, factors such as trauma, inflammation, hormonal fluctuations, and certain medications are believed to contribute to its development. The diagnosis of ILCH is accomplished through histopathological assessment and immunohistochemical staining, which aid in distinguishing it from other vascular lesions such as angiosarcoma, papillary endothelial hyperplasia, and deep venous thrombosis [9]. Additionally, other variations of ILCH, such as those affecting the corpus spongiosum and central nervous system, have also been documented [10]. While the exact treatment approach is not definitively established, surgical excision is recommended for managing pyogenic granuloma.

The objective of this review is to comprehensively present the clinical characteristics, origins, pathogenesis, diagnostic methods, treatment options, and outcomes associated with ILCH. The primary focus is on its intravascular origin and behavior. Through this scoping review, an in-depth understanding of the current knowledge about ILCH is provided.

## Review

Materials and methods

**Figure 1:**
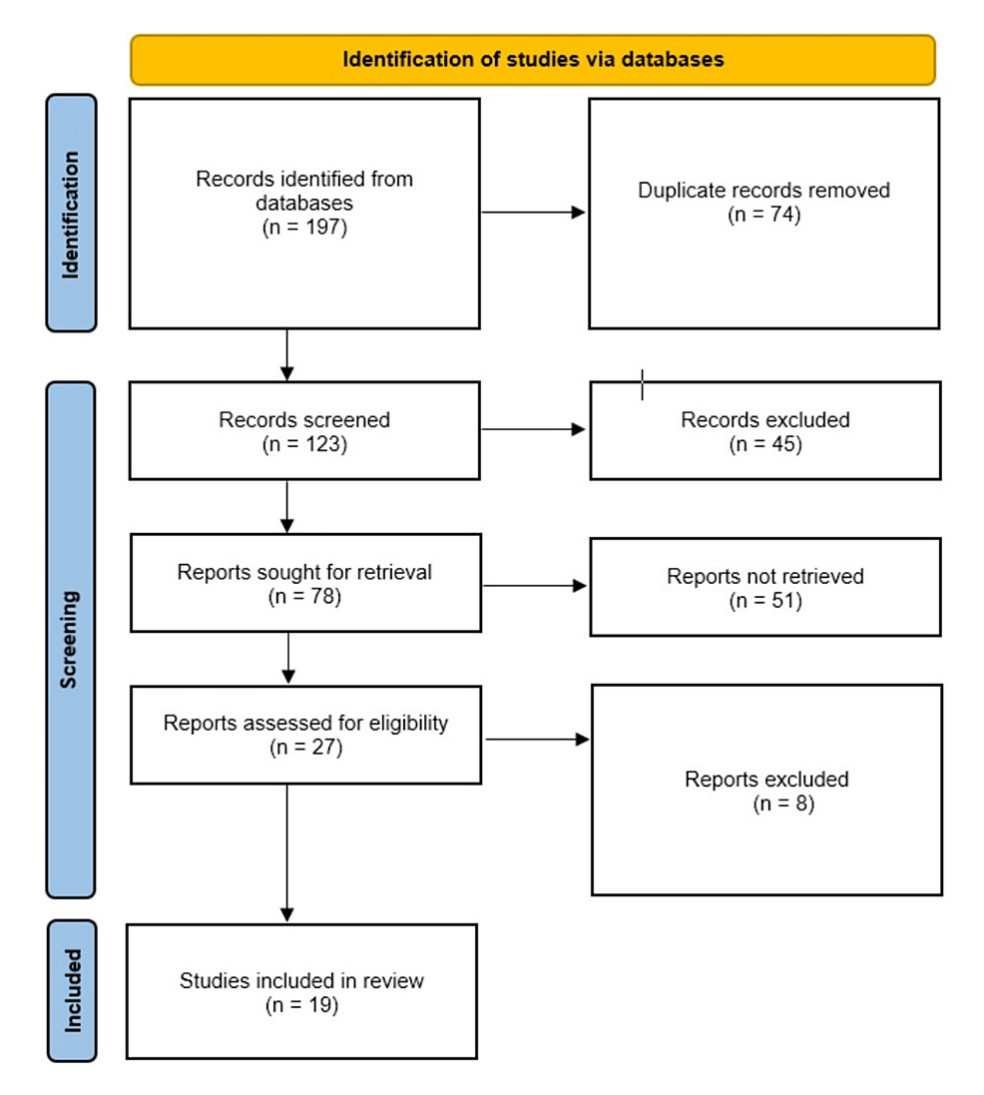
PRISMA flow diagram depicting the study selection process

This scoping review adhered to the PRISMA Extension for Scoping Reviews (PRISMA-ScR) guidelines [11]. Ethical committee approval was not required for this scoping review, as it involved the analysis of publicly available literature from online databases. Since this is a scoping review, it was not registered with the International Prospective Register of Systematic Reviews. A comprehensive search strategy was employed to identify relevant studies. Electronic databases, including PubMed, Web of Science, Cochrane, and Google Scholar, were searched for articles published up until June 25, 2023. A combination of relevant keywords and medical subject headings (MeSH terms) was used to optimize search results. The PRISMA-ScR flow diagram of the data search is shown below (Figure 1).

Inclusion criteria included peer-reviewed research articles, case reports, case series, and original articles. Studies focusing on ILCH or its synonyms, including articles discussing its clinical characteristics, origins, pathogenesis, diagnostic methods, treatment options, outcomes, and management, were also included. English-language articles were included to ensure comprehensive coverage. Exclusion criteria included non-peer-reviewed articles, reviews, editorials, letters to the editor, conference abstracts, and conference posters. Articles that did not primarily focus on ILCH or lack relevant information about its clinical characteristics, origins, pathogenesis, diagnostic methods, treatment options, outcomes, and management were also excluded. Articles in languages other than English due to potential limitations in translation and understanding were also excluded. These inclusion and exclusion criteria aim to ensure the selection of relevant and high-quality articles that contribute to the comprehensive understanding of ILCH.

The article screening process consisted of two stages. Initially, articles were screened based on their titles and abstracts by two independent reviewers (AG and HK) to determine their relevance as either relevant, not relevant, or potentially relevant. In the subsequent phase, the eligibility of full-text articles was assessed. SS and RI, acting independently, extracted data from the source publications onto a standard Microsoft Excel (Microsoft Corporation, Washington, United States) data extraction form. Following this, they individually applied predefined inclusion and exclusion criteria to identify potentially eligible studies. Any discrepancies that arose were subject to independent review by a third reviewer (MA), and resolutions were reached through collaborative discussion.

Results

An initial search using keywords yielded a total of 197 results. About 123 articles were selected for review by two independent reviewers after removing duplicates. After title and abstract screening, 78 articles were selected for full-text assessment, out of which 59 articles were further rejected based on exclusion criteria. A total of 19 articles were included in this review. This included 18 case reports and 1 cohort. The findings of the scoping review are presented using a narrative synthesis approach and are summarized in the following table (Table 1). Themes, patterns, and trends within the literature are discussed, providing a holistic understanding of ILCH. Seven articles are from the United States, three were from China, and one each from England, France, India, Japan, Korea, Spain, Switzerland, Taiwan, and the West Indies. There were a total of 59 participants including 29 males and 30 females. One hundred percent (n=59) underwent surgical excision and no recurrence was reported.

**Table 1:** The summary of findings of the selected studies USG: ultrasonography, MRI: magnetic resonance imaging, CT: computed tomography, MRV: magnetic resonance venography, PET: positron emission tomography, IJV: internal jugular vein, LCH: lobular capillary hemangioma, EJV: external jugular vein, SVC: superior vena cava, CTA: computed tomography angiography, DUS: Doppler ultrasound, ILCH: intravascular lobular capillary hemangioma

Author	Year	Location	Age	Gender	Diagnostic modality	Clinical findings	Intervention	Prognosis
Ulbright and Cruz [12]	1980	United States	12	Male	Physical examination	A 1.5-cm nontender, firm, and mobile nodule was palpated in the subcutaneous tissues of the right side of the neck. Histologically, the lesion displayed an intravascular projection on the vein wall's luminal side, comprising capillary proliferation with pronounced endothelial cells and myxoid stromal regions. Elastic fiber disruption was evident at its attachment. Electron microscopy emphasized capillary and stromal components.	Surgical excision	No recurrence was observed in the one-year follow-up.
Truong and Font [13]	1985	United States	44 and 68	Male	Biopsy	The features were mostly similar in both cases. Well-circumscribed, ovoid masses were easily removable from surrounding soft tissues and composed of discrete or confluent lobules of capillaries surrounded by fibromyxomatous stroma. Capillaries with variable sizes and round, oval, or angular lumina, mild nuclear hyperchromatism but no pleomorphism in the endothelial cell lining, and scattered mitotic figures were observed in endothelial cells. Poorly cellular intervening stroma with myxomatous matrix, collagen, reticulin fibers, and scattered lymphocytes; prominent vascular channels within the stroma, resembling small arteries or dilated venules. Absence of endothelial-lined papillary fronds, thrombi, surface ulcération, and granulation tissue. Non-invasion of the vein wall by the angiomatous proliferation.	Surgical excision	Case 1: The patient was reported to be in good health, without evidence of recurrence, seven years and eleven months after surgery. Case 2: The patient was alive and well, without evidence of recurrence, four years and seven months after surgical excision.
Pesce et al. [8]	1996	England	20	Male	Excisional biopsy	A cribriform, textured mass measuring 0.8x0.4 cm. Cellular stroma with plump fibroblasts and sparse lymphocytes. Slit-like luminal spaces were observed in some areas with thin endothelial lining. Mass adhered to the intima in some areas and was disrupted by capillary proliferation in the media in other areas. Parallel sections showed fibrous stroma with haphazardly arranged small vessels and a bland endothelial lining. Instances of capillaries abutting perineurial spaces were observed.	N/A	N/A
Margo [14]	1994	United States	27	Male	Biopsy	A muscular artery with a well-developed internal elastic membrane. Approximately one-third of the internal elastic membrane was replaced with fibrous connective tissue and disorganized smooth muscle cell proliferation. The lumen of the artery was filled with a polypoid mass of richly vascular tissue attached to the vessel wall. Plump and spindle-shaped endothelial cells with inconspicuous vascular lumina and sparse extracellular stroma. Presence of fibrin thrombi and chronic inflammatory cells, including eosinophils.	Surgical excision	N/A
Panchagnula and Kini [15]	2001	India	12	Female	Excisional biopsy	Interwoven thick and thin-walled blood vessels extend into the surrounding tissue. Intraluminal lobular masses, resembling "glomerulations," contained proliferating capillaries lined by prominent endothelial cells. FVIII antigen positivity.	Surgical excision	No recurrence was observed in the 1- to 1.5-year follow-up.
Hung et al. [4]	2004	Taiwan	44	Female	Excisional biopsy	A tender, dark-red to purplish eroded nodule was observed on the patient's right palm, featuring a hyperkeratotic collar and measuring 6x6 mm. Histological analysis revealed an elevated epidermal area containing multiple lobules of dilated and congested capillaries within vascular spaces in the papillary dermis. The epidermis displayed hyperkeratosis and papillomatosis. The deep dermis contained a collection of thick-walled vessels of varying caliber, largely lacking muscular elements. The Verhoeff-van Geison stain showed some areas of positive staining, suggesting an arteriole-like structure with elastic fibers.	Surgical excision	No recurrence was observed in the six-month follow-up.
Jung et al. [16]	2008	Korea	51	Male	USG, incisional biopsy	Clinical assessment revealed an oval subcutaneous mass measuring around 2×1 cm. Ultrasonography revealed a longitudinally elongated hypoechoic mass with limited inner vascularity within the cephalic vein. The tumor consisted of capillary lobules set in an oedematous fibromyxoid stroma, aligning with pyogenic granuloma.	Surgical excision	N/A
Pradhan et al. [6]	2008	United States	75	Female	CT scan, MRV, arteriography	CT scan showed an intraluminal tumor in the right internal iliac vein. MRV revealed an incompletely obstructing 2.4x1.8 cm mass in the right internal iliac vein, extending to the distal inferior vena cava. The arteriogram showed neovascularization of an intravenous mass. Intraoperative ultrasound showed the presence of a mass within the internal iliac vein extending into the inferior vena cava.	Surgical excision	No recurrence was observed in the two-month follow-up.
Barr and Vincek et al. [17]	2010	United States	45	Male	Physical examination, biopsy	Round, soft, moveable, deep dermal mass (1 cm in diameter) on the forehead. Histology showed a multilobulated tumor of capillaries separated by fibrous septae set in an edematous fibromyxoid stroma located in the deep subcutis, close to the frontalis muscle. CD31 staining performed to outline vessel distribution demonstrated the intravascular location of the lesion.	Surgical excision	N/A
Gamerio et al. [18]	2016	United States	54	Male	Incisional biopsy	Histology showed the proliferation of multiple well-circumscribed lobules (round-shaped or elongated) composed of packed capillary vessels lined by bland endothelial cells. A prominent population of pericytes surrounded the vessels.	Surgical excision	The lesion completely regressed within two weeks, and a disfiguring scar resulted from the regression.
Dermawan et al. [19]	2020	United States	13 to 85	Male(n=18) Female(n=22)	Biopsy	In ILCH cases, a distinct nodular or lobular growth pattern was evident, characterized by densely packed capillaries with central ectatic vessels. About half of the cases exhibited a clear peripheral vascular wall. Even in cases lacking visible peripheral walls, the well-defined growth pattern persisted. Multinodular growth was observed in some instances. Vascular spaces were lined by unremarkable endothelial cells, with hobnail features seen in 28% of cases. Approximately 25% displayed heightened mitotic activity (>5 mitotic figures/10 high-power fields), and mild cellular atypia was evident in 11% of cases. A significant inflammatory component was absent in the majority (75%) of cases. The presence of a pericyte layer encircling vessels was consistently highlighted by SMA staining. Notably, all cases exhibited diffusely positive WT1 staining in endothelial cells.	Surgical excision	The median follow-up period was 40 months (range: 7 to 153 months). Out of 21 cases, none of them recurred following surgical resection.
Balya et al. [20]	2021	France	35	Female	DUS, CTA, MRI, PET	DUS showed collateral vein dilatation. CTA revealed an intraluminal heterogeneous mass within the SVC, extending from the left brachiocephalic venous trunk to the distal third of the SVC. Thoracic MRI confirmed the intraluminal location of the SVC tumor, measuring 80x33 mm with a cystic component. Heterogeneous T1- and T2-weighted signals were evident. PET showed moderate fluorodeoxyglucose enhancement. Pathological examination demonstrated a tumor measuring 55x34x25 mm occupying the SVC lumen. Microscopic analysis unveiled a vascular lesion with lobular architecture, comprising capillaries lined by unstratified endothelium and fibro-oedematous stroma. Immunohistochemical analysis yielded positive vascular markers (CD31 and ERG) and a low Ki67 proliferation index.	Surgical excision	No recurrence was observed in the seven-month follow-up.
Ikeda et al. [21]	2021	Japan	72	Female	USG, CT, MRI	The left cephalic vein appeared enlarged. Ultrasound examination revealed a space-occupying lesion within the left subclavian vein, with no Doppler signal detected. The diameters of the left internal jugular vein were 6.0 mm (in a straight position) and 12.0 mm (during neck anteflexion), whereas the right internal jugular vein diameters were 3.0 mm (in a straight position) and 2.0 mm (during neck anteflexion). A potential venous thrombosis in the left subclavian vein was suspected, leading to the initiation of oral anticoagulant treatment. Following two weeks of treatment, ultrasonography indicated no change in the mass. The mass was surgically excised, and histopathological examination showed intravenous capillary haemangioma	Surgical resection	The sensation of pressure in the patient's left neck disappeared after the operation. The patient did not experience neck discomfort while cooking after discharge.
Jing et al. [2]	2021	China	38	Male	USG, biopsy	Ultrasound showed a well-defined nodular intraluminal mass within the basilic vein, measuring approximately 8x2x6 mm. A mainly hypoechoic and heterogeneous structure was observed, containing distinct anechoic tunnel-like structures. Abundant blood flow signals were evident on color Doppler flow imaging, supplied by surrounding tissue vessels. A pulsed Doppler examination recorded typical arterial signals within the lesion. A macroscopic examination revealed a well-defined polypoid mass connected to the venous wall. Microscopic analysis unveiled flattened endothelial cells lining capillaries in a lobular arrangement, separated by a fibrous mucinous stroma. Immunocytochemical staining confirmed vascularity with positive CD34 and ERG staining.	Surgical excision	No recurrence was observed in the three-month follow-up.
Kaiser et al. [22]	2021	Switzerland	26	Female	Excisional biopsy	Histopathological findings were typical of ILCH.	Surgical excision	No recurrence was observed in the three-month follow-up.
Tucciarone et al. [23]	2021	Spain	69	Female	CT scan, echography biopsy	Presence of a movable and non-pulsating mass along the EJV. An echography scan identified a 15x8 mm intraluminal lesion within the EJV. Doppler scanning revealed inherent arterial and venous blood flow. CT scan verified a non-occlusive intraluminal neoformation within the EJV. Subsequent histopathological examination definitively confirmed the presence of intravenous lobular capillary hemangioma.	Surgical excision	No recurrence was observed in the two-year follow-up.
Fakoory et al. [1]	2022	West Indies	47	Male	Biopsy, vascular USG	2x6 mm of soft, painless swelling along the trajectory of the temporal artery was identified. Vascular ultrasound identified thrombosis in the right temporal artery. Macroscopic analysis revealed multiple pieces of tan-gray tissue. Microscopic evaluation revealed a nodular vascular lesion characterized by small capillaries arranged in lobules. Immunohistochemistry demonstrated robust cytoplasmic positivity for CD31, CD34, and factor VIII.	Surgical excision	N/A
Zhou et al. [24]	2016	China	26	Female	USG, biopsy	A painless oval mass measuring 2x2 cm on the wrist's ulnar side. Ultrasound revealed an irregular low-echo area. Microscopic examination showed hypertrophic capillaries in clusters with clear borders and fibrous tissue in between, resembling LCH but without ulceration or inflammation. CD34, SMA, and FVIII positivity were observed.	Surgical excision	No recurrence was observed in the three-month follow-up.
Yang et al. [25]	2021	China	44	Male	Color Doppler USG, MRI, CT, biopsy	Doppler ultrasound revealed an abnormal echo within the left IJV, displaying hypoechoic patterns, irregular shape, and clear boundaries. Color Doppler showed a filling defect and microvascular imaging detected star-like flow signals. Contrast-enhanced USG demonstrated uneven enhancement during arterial and venous phases. CT and MRI confirmed an isodense pedunculated nodule in the IJV, ruling out thrombosis and suggesting endoluminal neoplasia. Immunohistochemical staining results showed CD34, CD31, ERG, VIM, and KI67 positivity with SMA negativity.	Surgical excision	No recurrence was observed in the three-month follow-up.

Discussion

ILCH is a subset of lobular capillary hemangioma (LCH) that presents a distinctive challenge due to its intravascular origin and rarity. This rare subset, now recognized as IVLCHs by the International Society for the Study of Vascular Anomalies, remains an uncommon topic within the vascular pathology literature [26]. The scarcity of documented cases underscores the unique nature of ILCHs. Although instances of IVLCHs are not confined to any specific racial group, they have been observed to be slightly more prevalent among women, children, and young adults [1,2,26].

ILCHs often present a diagnostic challenge due to their diverse clinical manifestations and potential asymptomatic nature. Presenting symptoms can range from localized swelling to discomfort or pain. However, their rarity and subtle clinical signs can often lead to delayed recognition. The exact pathogenesis of ILCH remains elusive, with congenital endothelial hyperplasia considered a contributing factor [27]. While the majority of cases occur sporadically, associations with trauma, infections, and hormonal changes have been proposed. Recent studies have also highlighted the involvement of both sprouting angiogenesis (SA) and intussusceptive angiogenesis (IA) in ILCH formation, a mechanism previously only attributed to SA [28,29].

Histologically, ILCHs exhibit a distinctive lobular architecture marked by unstratified endothelium forming capillaries within an edematous fibromyxoid stroma [20]. These lobules are separated by venular structures, emphasizing the lesion's multi-venular origin. Characteristic features such as capillary-sized vessels and interendothelial contacts distinguish ILCH from other vascular tumors. The diagnostic challenge lies in its resemblance to other entities such as venous thrombosis, papillary endothelial hyperplasia, atypical vascular proliferation, histiocytoid hemangioma, and angiosarcoma [30]. Accurate differentiation is crucial due to the potentially fatal implications of misdiagnosing malignant intravascular tumors. Various imaging techniques aid in ILCH diagnosis, including ultrasound, MRI, and CT [6,20,25]. Ultrasound offers insights into vascular patterns, while MRI and CT provide anatomical details for surgical planning [2]. However, histopathological examination remains essential for definitive diagnosis.

The primary approach to managing ILCH is surgical excision, a step that not only confirms the diagnosis but also prevents potential complications [4,23,26,30]. These complications encompass occlusion of the superior vena cava (SVC), thrombosis, localized compression, and the risk of pulmonary embolization. Nevertheless, resecting the SVC is an infrequent procedure, and deliberations persist concerning the optimal approach for graft replacement. A primary concern associated with graft insertion is the likelihood of thrombosis, often manifesting within one to five months following implantation. The endurance of graft patency over the long-term hinges on diverse factors, including the choice of graft material, its length, and configuration. Options encompass the utilization of expanded polytetrafluoroethylene (ePTFE) or biological grafts like bovine pericardium [31]. In a study executed by Maurizi et al. in 2019, the researchers detailed their experiences with SVC reconstruction using both bovine pericardial conduit and ePTFE grafts [32]. Their findings demonstrated no statistically discernible variance in graft patency based on the type of graft employed. In the case under consideration, an ePTFE vascular graft fashioned into an L-shaped structure was chosen. Additionally, the implementation of a left brachiobasilic fistula aimed to amplify blood flow and mitigate the likelihood of graft thrombosis. Despite the favorable long-term outlook attributed to the benign character of ILCH, immediate postoperative anticoagulant treatment is of paramount importance. Furthermore, vigilant monitoring of the graft's patency through regular Doppler ultrasound examinations is imperative to ensure its sustained functionality [33,34].

The study of ILCH is still in its infancy, but the potential for further exploration and understanding is vast. As medical knowledge continues to advance, there are several avenues of future research that could shed light on this enigmatic vascular anomaly. The underlying molecular mechanisms driving the formation of ILCHs remain largely unknown. Investigating the genetic and molecular alterations within these lesions could provide insights into their origin and growth patterns. Identifying potential biomarkers specific to ILCHs could aid in their early detection and differentiation from other intravascular lesions. While current imaging techniques like ultrasound, MRI, and CT are valuable tools, the development of more advanced imaging modalities could enhance diagnostic accuracy. Molecular imaging approaches that target specific cellular and molecular markers associated with ILCHs might provide real-time visualization and improved characterization.

Given the rarity of ILCHs, building comprehensive patient registries could facilitate data collection, analysis, and collaborative research efforts. Sharing clinical and pathological data among healthcare institutions could help uncover patterns, prognostic factors, and treatment outcomes. As our understanding of the molecular basis of ILCHs deepens, the potential for targeted therapies could emerge. Developing therapies that specifically target the pathways driving ILCH growth and proliferation might offer non-invasive treatment options, minimizing surgical interventions and associated complications. Comprehensive long-term follow-up studies of patients who have undergone surgical resection or other treatment approaches for ILCHs are essential. These studies could provide insights into the recurrence rate, long-term patency of grafts in cases of SVC reconstruction, and potential late complications. Further exploration of surgical techniques and approaches for the removal of ILCHs could lead to improved patient outcomes. Research focusing on minimally invasive procedures, robotic-assisted surgery, and endovascular interventions could reduce postoperative complications, shorten recovery times, and enhance cosmetic outcomes.

Investigating the genetic and genomic alterations associated with ILCHs could provide valuable insights into their etiology and pathogenesis. Whole-genome sequencing and comparative genomic analyses could uncover genetic mutations or alterations specific to ILCHs. Developing animal models that mimic the formation and progression of ILCHs could provide a platform for preclinical research. These models could be used to study the impact of various interventions, test potential therapies, and unravel the complex cellular and molecular processes underlying ILCH development. Given the rarity of ILCHs, collaboration between institutions and researchers on a global scale is essential. International collaborations could pool data, resources, and expertise, accelerating research progress and leading to a more comprehensive understanding of ILCHs. Long-term studies focusing on patient outcomes and quality of life following surgical resection or other treatments are crucial. Understanding the physical, psychological, and functional impacts of ILCHs on patients could guide personalized treatment approaches and postoperative care strategies.

## Conclusions

In conclusion, this scoping review provides an in-depth exploration of the rare and distinct subset of LCH known as ILCH. The review synthesizes existing literature to comprehensively examine ILCH's clinical characteristics, origins, pathogenesis, diagnostic methods, treatment options, and outcomes. Despite its rarity, ILCH poses unique diagnostic and management challenges due to its intravascular location. Accurate differentiation from other vascular lesions is crucial, highlighting the necessity for histopathological confirmation. The review underscores ILCH's clinical presentation, histological architecture, imaging techniques, and diagnostic tools. Surgical excision is the primary treatment due to potential complications, such as SVC occlusion and thrombosis. The article suggests future research directions, including molecular mechanisms, targeted therapies, patient registries, and minimally invasive techniques. Emphasis on international collaboration and long-term outcome studies is vital to advancing our understanding of this enigmatic vascular anomaly.
